# Adjunctive Thrombolytics After Successful Endovascular Reperfusion: A Systematic Review and Meta‐Analysis of Randomized Controlled Trials

**DOI:** 10.1002/ana.70021

**Published:** 2025-08-13

**Authors:** Mohamed F. Doheim, Mahmoud H. Mohammaden, Ammar Jumah, Qingwu Yang, Wenjie Zi, Yangmei Chen, Yamei Tang, Yajie Liu, Xiaochuan Huo, Liping Liu, Bernard Yan, Zhongrong Miao, Wei Hu, Chunrong Tao, Xinfeng Liu, Liqun Jiao, Xuesong Bai, Wenhuo Chen, Diogo C. Haussen, Thanh Nguyen, Raul G. Nogueira

**Affiliations:** ^1^ UPMC Stroke Institute, Department of Neurology and Neurosurgery University of Pittsburgh School of Medicine Pittsburgh PA USA; ^2^ Department of Neurology Grady Memorial Hospital, Emory University School of Medicine Atlanta GA USA; ^3^ Department of Neurology Xinqiao Hospital and The Second Affiliated Hospital, Army Medical University (Third Military Medical University) Chongqing People's Republic of China; ^4^ Department of Neurology The Second Affiliated Hospital of Chongqing Medical University Yuzhong District People's Republic of China; ^5^ Department of Neurology Sun Yat‐sen Memorial Hospital, Sun Yat‐sen University Guangzhou People's Republic of China; ^6^ Department of Neurology Shenzhen Hospital of Southern Medical University Shenzhen People's Republic of China; ^7^ Cerebrovascular Disease Department, Neurological Disease Center Beijing Anzhen Hospital, Capital Medical University Beijing People's Republic of China; ^8^ China National Clinical Research Center for Neurological Diseases Beijing Tiantan Hospital, Capital Medical University Beijing People's Republic of China; ^9^ Department of Neurology at Melbourne Brain Center The University of Melbourne Medicine at Royal Melbourne Hospital Parkville Victoria Australia; ^10^ Interventional Neuroradiology, Department of Neurology Beijing Tiantan Hospital, Capital Medical University Beijing People's Republic of China; ^11^ Department of Neurology, The First Affiliated Hospital of USTC, Division of Life Sciences and Medicine University of Science and Technology of China Hefei People's Republic of China; ^12^ Department of Neurosurgery and Interventional Neuroradiology Xuanwu Hospital, China International Neuroscience Institute, Capital Medical University, National Center for Neurological Disorders Beijing People's Republic of China; ^13^ Department of Neurosurgery Xuanwu Hospital, China International Neuroscience Institute, Capital Medical University, National Center for Neurological Disorders Beijing People's Republic of China; ^14^ Department of Cerebrovascular Disease Fujian Medical University Union Hospital Fuzhou People's Republic of China; ^15^ Department of Neurology Boston Medical Center, Chobanian and Avedisian School of Medicine Boston MA USA

## Abstract

**Objective:**

The efficacy and safety of intra‐arterial thrombolysis (IAT) as an adjunct to endovascular thrombectomy (EVT) in large vessel occlusion strokes (LVOS) remain uncertain, with recent randomized controlled trials (RCTs) yielding conflicting results. This meta‐analysis aimed to assess the impact of IAT following successful EVT in patients with LVOS.

**Methods:**

A comprehensive search was conducted across PubMed, ClinicalTrials.gov, the Cochrane Library databases, and the International Stroke Conference 2025 abstracts to identify RCTs evaluating IAT following successful EVT from January 2015 to February 2025. The primary outcome was the odds of achieving an excellent functional outcome (defined as a modified Rankin Scale [mRS] = 0–1 at 90 days). Secondary outcomes included 90‐day functional independence (mRS = 0–2). Safety measures included symptomatic intracranial hemorrhage (sICH), and mortality at 90 days. The protocol was registered in PROSPERO (CRD420250651602).

**Results:**

The primary pooled analysis of 6 RCTs (*N* = 1,974) showed that IAT significantly increased the likelihood of achieving excellent functional outcome at 90 days (mRS = 0–1: odds ratio [OR] = 1.47, 95% confidence interval [CI] = 1.21–1.80, *p* < 0.001), with a notable effect in anterior circulation (OR = 1.48, 95% CI = 1.18–1.87, *p* < 0.001) but not in posterior circulation LVOS (OR = 1.51, 95% CI = 0.83–2.74, *p* = 0.18). Among thrombolytic drugs, alteplase was most strongly associated with favorable outcomes (mRS = 0–1: OR = 1.94, 95% CI = 1.31–2.87, *p* < 0.001), followed by tenecteplase (OR = 1.43, 95% CI = 1.08–1.89, *p* = 0.01). No significant safety concerns were observed, as there was no increase in the odds of sICH (OR = 1.15, 95% CI = 0.75–1.75, *p* = 0.51) or 90‐day mortality (OR = 1.00, 95% CI = 0.79–1.26, *p* = 0.99). Sensitivity analyses for all outcomes yielded consistent results.

**Interpretation:**

IAT following successful EVT significantly enhances the likelihood of achieving an excellent functional outcome, particularly in anterior circulation strokes. Although the benefit in posterior circulation strokes remains uncertain, the lack of significant differences in sICH risk and mortality across thrombolytic drugs and stroke locations support the safety of IAT. ANN NEUROL 2025;98:1299–1314

Endovascular thrombectomy (EVT) is the standard treatment for large vessel occlusion stroke (LVOS) in both the anterior and posterior circulations.^1^, ^2^, ^3^, ^4^ Despite high rates of successful recanalization (typically defined as modified or expanded thrombolysis in cerebral infarction [TICI] 2b‐3, achieved in approximately 80% or more of cases), a significantly smaller proportion of patients—less than 50%—achieve favorable functional outcomes at 90 days.^5^, ^6^, ^7^, ^8^ This paradox may result from a complex interaction of factors, including pre‐existing tissue infarction, the multidimensional composition of clots, the integrity of collateral circulation, downstream microvascular injury, and medical complications after stroke. Even after successful recanalization, these elements influence reperfusion efficacy and clinical outcomes.^5^, ^6^, ^9^, ^10^

The interplay between macro‐ and microcirculation, often termed the “no‐reflow phenomenon,” presents a potential therapeutic target for intra‐arterial thrombolysis (IAT). At the macrovascular level, IAT with alteplase has been shown to potentially enhance functional outcomes by facilitating reperfusion and improving its grading in the early days, before advancements in EVT reshaped stroke treatment.^11^, ^12^ Meanwhile, persistent microthrombi may impede recovery at the microvascular level despite normal or near‐normal angiographic findings.^5^, ^9^, ^10^ Emerging evidence suggests that combining IAT with EVT could enhance functional outcomes by targeting residual microvascular thrombi, thereby optimizing cerebral perfusion.^9^, ^13^, ^14^

In a survey of 104 neurointerventionalists, over 60% used IAT with EVT, but consensus on its indication and efficacy was lacking. Among users, 60.4% applied it for distal occlusions, rescue therapy, or as an adjunct. Whereas 49.4% called for more evidence, 37.6% supported its utility, and only 12.9% dismissed its relevance in modern practice.^15^ IAT is commonly used as an adjunct or rescue therapy in LVOS, yet its benefit following successful EVT remains uncertain, with prior meta‐analyses yielding conflicting results, likely due to their retrospective design.^13^, ^16^ These studies often did not distinguish between successful and unsuccessful reperfusion before IAT or differentiate its use as a rescue versus adjunct therapy, complicating intention‐to‐treat analyses. Additionally, the inclusion of studies incorporating antiplatelet glycoprotein IIb/IIIa inhibitors contributed to heterogeneity.^13^ Furthermore, randomized controlled trials (RCTs) focusing solely on successful recanalization have varied in their inclusion criteria and therapeutic drugs, and produced mixed results.^17^, ^18^, ^19^, ^20^, ^21^, ^22^ This systematic review and meta‐analysis (Adjunctive Thrombolytics After Successful Endovascular Reperfusion [ATLAS‐ER]) aimed to assess the potential benefit and safety of IAT after successful EVT in patients with LVOS. It focused exclusively on data from RCTs across various locations, reperfusion TICI scores, and therapeutic drugs to understand both the microcirculatory and macrocirculatory effects of IAT.

## Methods

This systematic review and meta‐analysis followed the methodology outlined in the Cochrane Handbook for Systematic Reviews of Interventions.^23^ The study adhered to the Preferred Reporting Items for Systematic Reviews and Meta‐Analyses (PRISMA) guidelines to ensure transparent and standardized reporting.^24^ The review protocol was prospectively registered in the PROSPERO database under registration number CRD420250651602.

### Search Strategy

A comprehensive search was performed across PubMed, ClinicalTrials.gov, Cochrane Library, and Web of Science databases, covering all records from January 2015 to February 2025. The search strategy combined multiple keywords and phrases to capture relevant literature, including terms such as “acute ischemic stroke,” “ischemic stroke,” “stroke,” “large vessel occlusion,” “brain ischemia,” “cerebral infarction,” “middle cerebral artery,” “mechanical thrombectomy,” “endovascular therapy,” “thrombolytics,” “alteplase,” “tenecteplase,” “tissue plasminogen activator,” “intra‐arterial thrombolysis,” “rtPA,” and “urokinase,” among others. Additional methods included expert consultations, conference presentations, manual screening of relevant studies and presentations at the International Stroke Conference (ISC) in February 2025, and review of clinical trial registries to ensure both published and unpublished data from trials were included.

### Eligibility Criteria and Screening

Inclusion criteria were restricted to RCTs that involved adult patients (≥ 18 years old) receiving IAT after successful EVT for LVOS. Exclusion criteria included studies lacking a control group, those involving non‐randomized observational designs, and the use of IAT as a rescue rather than adjunct treatment. Titles, abstracts, and full‐text articles were screened against these eligibility criteria by two independent reviewers (authors M.F.D. and M.H.M.), with disagreements resolved through discussion with the senior author (R.G.N.) as needed.

### Data Extraction

Two independent investigators (authors M.F.D. and A.J.) extracted data on baseline characteristics, primary, secondary, and safety endpoints from each eligible study to ensure accuracy and consistency. Data extraction focused on structured reports, which included details such as study sites, trial identifiers, and the number of patients screened. They also detailed the number of patients included in the IAT and control groups, along with baseline demographic characteristics such as age, sex (male and female), and location. Information on intravenous thrombolysis (IVT) before randomization, the IAT dose, and the time window was included. Additionally, the reports provided data on the time from stroke onset to the start of study treatment (National Institutes of Health Stroke Scale [NIHSS] score and Alberta Stroke Program Early CT Score [ASPECTS]), and the type of baseline imaging used. The expanded thrombolysis in cerebral infarction (e‐TICI) scores, primary outcomes, and results, along with effect sizes and their 95% confidence intervals (CIs), were extracted.

### Outcomes

The primary outcome was an excellent functional outcome at 90 days, defined as a modified Rankin Scale (mRS) score of 0 to 1. Secondary outcomes included functional independence defined as mRS = 0 to 2 at 90 days. Safety measures included symptomatic intracranial hemorrhage (sICH), as defined by each study, and 90‐day mortality.

### Assessment of Quality and Risk of Bias

The Cochrane Risk of Bias (RoB2) tool was used to assess the quality and potential biases of the included RCTs.^25^ This tool evaluates risk of bias across 5 domains: (1) randomization process, (2) deviations from intended interventions, (3) missing outcome data, (4) outcome measurement, and (5) selection of the reported result. Each domain was rated as low, high, or some concerns based on the available study information. Two independent reviewers (authors M.F.D. and M.H.M.) conducted this assessment, and discrepancies were resolved through discussion, with input from the senior author (R.G.N.) as needed. The quality of evidence was assessed independently by 2 reviewers (authors M.F.D. and M.H.M.) using the Grading of Recommendations, Assessment, Development, and Evaluation (GRADE) framework, which evaluates domains including risk of bias, inconsistency, indirectness, imprecision, and publication bias. Evidence was categorized as very low, low, moderate, or high certainty.^26^

### Statistical Analysis

Effect sizes were calculated as logit‐transformed odds ratios (ORs) using random effects models with Mantel–Haenszel weighting. Between‐study variance was estimated using restricted maximum likelihood, with 95% CIs. Subgroup analyses explored sources of heterogeneity, such as IAT type (alteplase vs tenecteplase vs urokinase), TICI scores at randomization (TICI 2b‐3 vs TICI 2c‐3 vs TICI 3), prior IVT administration, and location (anterior vs posterior). Heterogeneity was assessed using the Q statistic and *I* ^2^ test, with significant heterogeneity indicated by *I* ^2^ ≥ 50% or a *p* value < 0.10 from the *Q* test. Due to the small number of included studies (< 10), publication bias was not assessed, and meta‐regression was not performed. Sensitivity analyses were conducted by sequentially excluding one study at a time to assess the robustness of the findings.^23^, ^27^, ^28^ Statistical analyses were performed using Stata software (version 17.0; StataCorp LLC, College Station, TX, USA).

## Results

### Search Results

A total of 3,517 potential studies were identified. After removing 1,120 duplicates, 2,397 studies remained for further screening. After the title and abstract review, 33 studies proceeded to full‐text screening. Following a thorough examination, 6 RCT studies were included for primary analysis, including 4 published RCTs^17^, ^18^, ^19^, ^20^ and 2 RCTs (the ANGEL‐TNK and PEARL trials) presented at the 2025 International Stroke Conference^21^, ^22^ (Supplementary Fig S1). The DATE trial was a 2‐part, phase Ib/IIa, multicenter, open‐label study in China evaluating the safety of adjunctive tenecteplase following successful EVT (eTICI ≥ 2b50 within 24 hour of last known feeling well). In part Ib, up to 4 escalating doses (starting at 0.03125 mg/kg to a planned maximum of 0.1875 mg/kg) were tested with sICH within 24 hours as the primary outcome. In part IIa, 157 patients were randomized to 2 selected doses (0.03125 and 0.0625 mg/kg) or placebo. As the trial primary focus was safety rather than efficacy, it was excluded from our main analysis. However, we conducted a secondary analysis, pooling data from part IIa to explore efficacy signals.^29^

### Study Characteristics and Risk of Bias Assessment

The baseline characteristics across the studies varied, but key patterns were observed. The median age ranged from 65.8 to 72 years, with most studies showing a male predominance (54% to 75.5%). The stroke location was primarily in the internal carotid artery (ICA), middle cerebral artery (MCA) M1, or M2 segments in the majority of studies, with one study (ATTENTION‐IA) focusing on the posterior circulation (vertebral, basilar, and posterior cerebral artery [PCA]‐P1 segment).^19^ The IAT drugs varied among trials between tenecteplase (3 RCTs), alteplase (2 RCTs), and urokinase (1 RCT). Perfusion imaging, such as computed tomography perfusion (CTP) and/or perfusion‐weighted imaging (PWI) was used in some trials,^20^, ^22^ whereas others did not utilize advanced imaging. All studies included in this analysis were multicenter RCTs comparing adjunctive IAT with best medical management or placebo following successful EVT (defined as eTICI ≥ 2b50) in patients with acute ischemic stroke due to LVO treated within 24 hours of last known feeling well. Regarding the choice of intra‐arterial thrombolytic agents, the CHOICE and PEARL trials used alteplase at a dose of 0.225 mg/kg, whereas the POST‐TNK, ATTENTION‐IA, and ANGEL‐TNK trials used tenecteplase at doses up to 0.125 mg/kg. The POST‐UK trial was unique in using IA urokinase at a dose of 100,000 IU. With respect to reperfusion status before randomization, POST‐TNK and POST‐UK included only patients with near‐complete to complete reperfusion (eTICI 2c/3), whereas the other trials also included patients with eTICI 2b50/67 after EVT. Finally, IVT prior to EVT was permitted in the CHOICE, ATTENTION‐IA, and PEARL trials but was not allowed in the other studies (see the Table 1). All included RCTs were of good quality and low risk of bias (Supplementary Tables S1 and S2).

**Table 1:** Baseline Characteristics and Treatment Metrics of Randomized Clinical Trials on Intra‐Arterial Thrombolysis After Mechanical Thrombectomy

	Choice^20^	Post‐TNK^18^	Post‐UK^17^	Angel‐TNK^22^	Attention‐IA^19^	PEARL^21^	DATE^29^
Study information							
Author/year	Renú/2022	Huang/2025	Liu/2025	Huo/2025	Hu/2024	Yang/2025	Hou/2025
Study sites	7 Spain	34 China	35 China	19 China	31 China	28 China	30 China
Identifier	NCT03876119	ChiCTR2200064809	ChiCTR2200065617	NCT05624190	NCT05684172	NCT05856851	ChiCTR2400080624
Included	113	540	534	255	208	324	157
IAT: control	61:52	269:271	267:267	126:129	104:104	164:160	92:65
Baseline demographic characteristics							
Age, yr	Mean: 70.6	Median: 69 yr	Median: 69	Median: 72	Mean: 66	Mean: 65.8	Median: 71
Female	52 (46%)	221 (41%)	223 (41.8%)	114 (44.7%)	51 (24.5%)	99 (30.6%)	68 (43.3%)
Location	ICA‐T, MCA‐M1 or M2	ICA, MCA‐M1 or M2	ICA, MCA‐M1 or M2	ICA, MCA‐M1 or M2	Vertebral, basilar and PCA‐P1	ICA‐T, MCA‐M1 or M2	ICA, M1, or M2
IVT (before randomization)	IAT: 38 (62%) Placebo: 31 (60%)	No	No	No	IAT: 28 (26.9%) Placebo: 25 (24%)	IAT: 69 (42.1%) Placebo: 66 (41.3%)	No
IAT drug	tPA	TNK	Urokinase	TNK	TNK	tPA	TNK
Dose	0.225 mg/kg	0.0625 mg/kg	100,000 IU	0.125 mg/kg	0.0625 mg/kg	0.225 mg/kg	0.03125 or 0.0625 mg/kg
Time from onset to treatment, min	315.0 (218–680)	504 (310–760)	529 (322–787)	588 (399–841.2)^a^	420 (276–564)^a^	438.5 (271.5–709)	417 (263–761)
Stroke severity							
NIHSS, median (IQR)	IAT: 14 (8–20) Placebo: 14 (10–20)	IAT: 15 (11–20) Placebo: 15 (10–20)	IAT: 15 (11–19) Placebo: 15 (10–19)	IAT: 15 (12–19) Placebo: 16 (12–19)	IAT: 19.5 (12–35) Placebo: 23 (14–35)	IAT: 15 (11–17) Placebo: 15 (11–18)	IAT: 17 (12–20) or 17 (10–20) Placebo: 17 (12–20)
ASPECTS, median (IQR)	IAT: 9.0 (9.0–10.0) Placebo: 10.0 (8.0–10.0)	IAT: 8 (7–9) Placebo: 8 (7–9)	IAT: 8 (7–9) Placebo: 8 (7–9)	IAT: 7 (6–8) Placebo: 7 (6–8)	IAT: 9 (8–10)^b^ Placebo: 8 (8–10)	IAT: 9 (7–10) Placebo: 9 (7–10)	IAT:8 (6–9) or 9 (8–9) Placebo: 8 (7–9)

aRR = adjusted risk ratio; ASPECTS = Alberta Stroke Program Early CT Score; CI = confidence interval; CTP = computed tomography perfusion; eTICI = expanded Treatment in Cerebral Ischemia; EVT = endovascular thrombectomy; IAT = intra‐arterial thrombolysis; ICA‐T = intracranial artery terminus; ICH = P1 = main trunk of the posterior cerebral artery; IQR = interquartile range; IU = international unit; IVT = intravenous thrombolysis; M1 = main trunk of the middle cerebral artery; M2 = first order branch of the middle cerebral artery; MCA = middle cerebral artery; mRS = modified Rankin Scale; N/A = not applicable; NIHSS = National Institutes of Health Stroke Scale; PWI = perfusion weighted image; intracranial hemorrhage; SD = standard deviation; TNK = tenecteplase; tPA = tissue plasminogen activator; UK = urokinase.

^a^ Onset to randomization, (hours were converted to minutes).

^b^ Posterior ASPECT score.

## Excellent Functional Outcome (mRS = 0–1) at 90 Days

**FIGURE 1:**
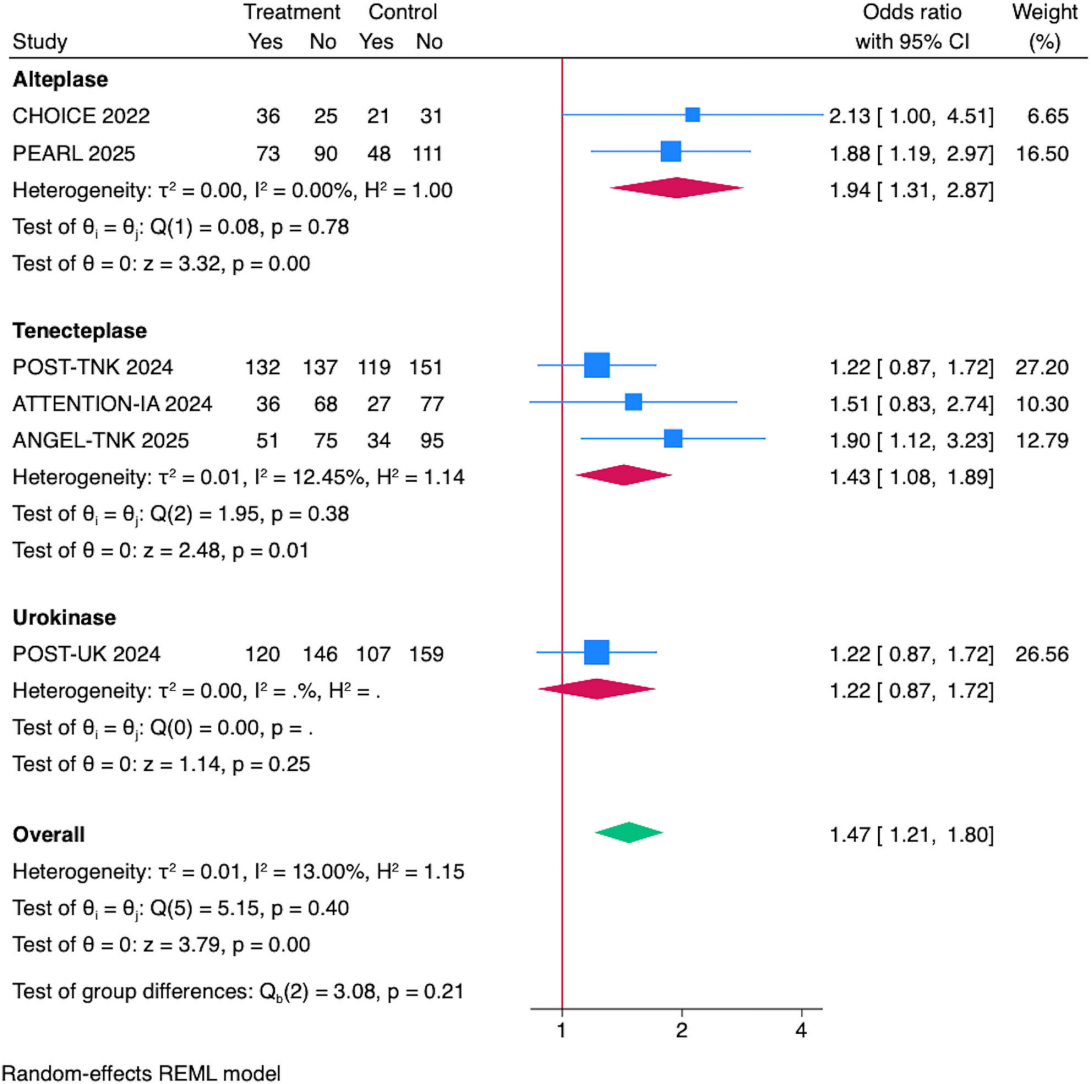
Association between IAT and functional outcomes at 90 days. Pooled analysis showed significant improvement in mRS = 0–1 with IAT (OR = 1.47, 95% CI = 1.21–1.80, *p* < 0.001; *I* ^2^ = 13%). Subgroup analysis showed significant benefit with alteplase (OR = 1.94, 95% CI = 1.31–2.87, *p* < 0.001) and tenecteplase (OR = 1.43, 95% CI = 1.08–1.89, *p* = 0.01), but not urokinase (OR = 1.22, 95% CI = 0.87–1.72, *p* = 0.25). CI = confidence interval; IAT = intra‐arterial thrombolysis; mRS = modified Rankin Scale; OR = odds ratio. [Color figure can be viewed at www.annalsofneurology.org]

The pooled analysis of all included RCTs demonstrated a significant association with excellent functional outcome (mRS = 0–1) at 90 days (OR = 1.47, 95% CI = 1.21–1.80, *p* < 0.001) with low heterogeneity (*I* ^2^ = 13.00%; Fig 1). Sensitivity analyses, including leave‐one‐out evaluations, confirmed the robustness of the results, with no single trial disproportionately influencing the pooled effect size or heterogeneity (Supplementary Fig S2A). Sensitivity analyses excluding RCTs presented at ISC 2025 and including data from the DATE trial (phase 2a) confirmed the robustness of the results (Supplementary Figs S3A and S4A). The overall combined distribution of mRS scores at 90 days (CHOICE, POST‐TNK, POST‐UK, ANGEL‐TNK, and PEARL trials) is described in Supplementary Figure S5A. The pooled common odds ratio (cOR) for improved functional outcomes with IAT was 1.22 (95% CI = 1.05–1.42), indicating a significant benefit with no observed heterogeneity (*I* ^2^ = 0%).

### Subgroup Analysis by IAT Drug Type

A subgroup analysis stratified by the IAT drug showed significant improvement in excellent functional outcome (mRS = 0–1) at 90 days in those who received alteplase (OR = 1.94, 95% CI = 1.31–2.87, *p* < 0.001) with low heterogeneity (*I* ^2^ = 0.00%). Similarly, tenecteplase showed significant benefit (OR = 1.43, 95% CI = 1.08–1.89, *p* = 0.01) with low heterogeneity (*I* ^2^ = 12.45%). In contrast, urokinase, represented by only one RCT; the POST‐UK trial, did not show a significant effect (OR = 1.22, 95% CI = 0.87–1.72, *p* = 0.25; see Fig 1).

### Subgroup Analysis by Stroke Location

**FIGURE 2:**
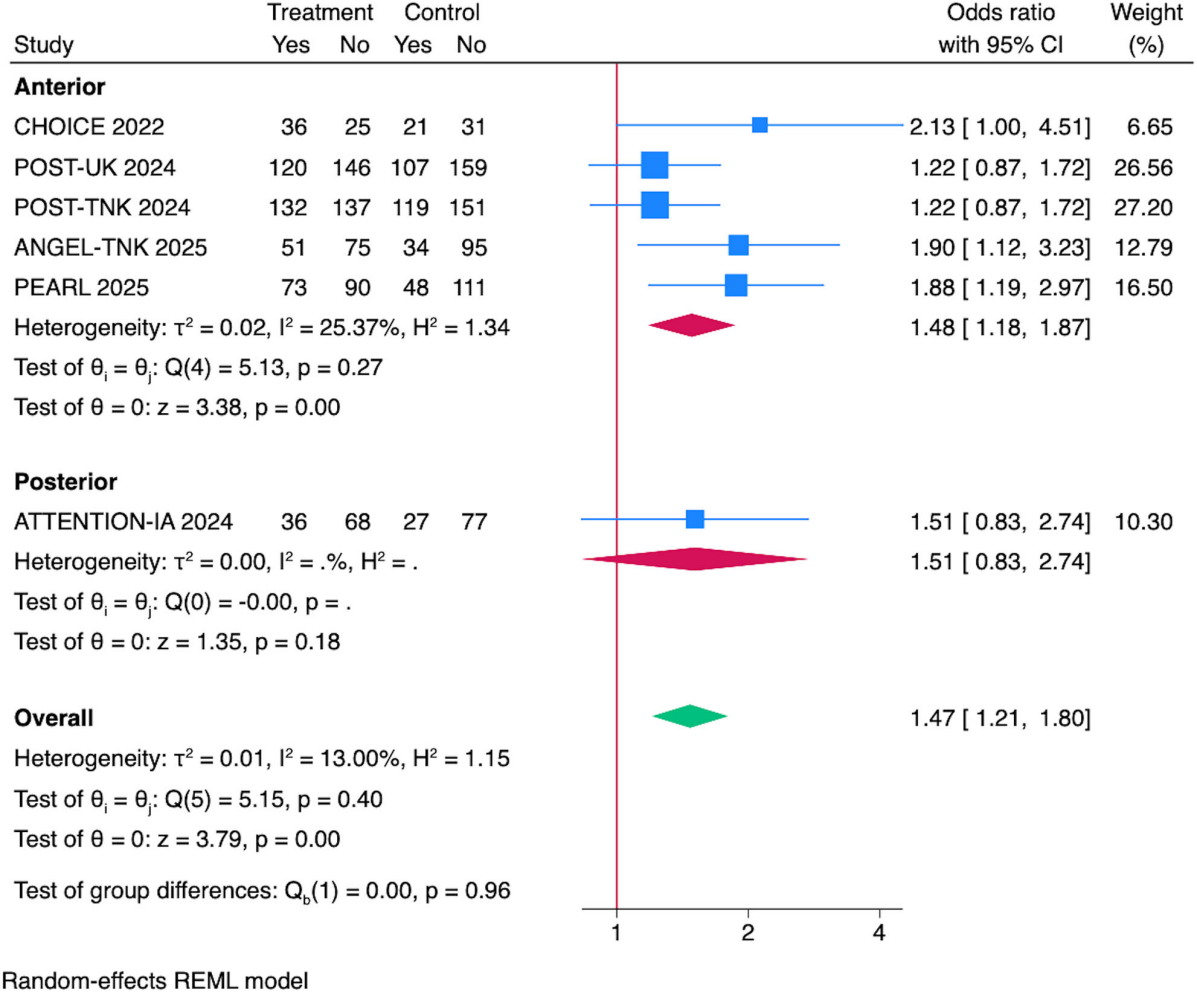
Association between IAT and functional outcomes at 90 days. Pooled analysis of all RCTs showed a significant improvement in mRS = 0–1 with IAT (OR = 1.47, 95% CI = 1.21–1.80, *p* < 0.001; *I* ^2^ = 13%). Subgroup analysis showed significant benefit for anterior circulation strokes (OR = 1.48, 95% CI = 1.18–1.87, *p* < 0.001) but not for posterior circulation strokes (OR = 1.51, 95% CI = 0.83–2.74, *p* = 0.18). CI = confidence interval; IAT = intra‐arterial thrombolysis; mRS = modified Rankin Scale; OR = odds ratio; RCTs = randomized controlled trials. [Color figure can be viewed at www.annalsofneurology.org]

A subgroup analysis by stroke location revealed a significant benefit in anterior circulation strokes (OR = 1.48, 95% CI = 1.18–1.87, *p* < 0.001), whereas the effect in posterior circulation strokes with only one RCT included (ATTENTION‐IA) was not statistically significant (OR = 1.51, 95% CI = 0.83–2.74, *p* = 0.18; see Fig 2).

### Subgroup Analysis by Trial Inclusion Reperfusion Criteria

Compared with RCTs that included only patients with eTICI 2c‐3, those that included eTICI 2b50‐3 demonstrated a stronger association with treatment success. Including ATTENTION‐IA in the analysis resulted in an OR of 1.83 (95% CI = 1.38–2.41, *p* < 0.001; Supplementary Fig S6A), indicating a significant benefit. After excluding ATTENTION‐IA, focusing on anterior circulation only, the OR increased slightly to 1.93 (95% CI = 1.41–2.64, *p* < 0.001). In contrast, for the eTICI 2c‐3 subgroup, the OR was insignificant at 1.22 (95% CI = 0.96–1.56, *p* = 0.10; Supplementary Fig S6B).

### Subgroup Analysis by eTICI 2c‐3 versus 3

To assess the impact of IAT on microcirculation “no‐reflow phenomenon,” we conducted subgroup analyses following near‐complete (TICI 2c‐3) or complete reperfusion (TICI 3). For the TICI 3 subgroup, a meta‐analysis of 2 RCTs (POST‐UK and POST‐TNK) including 2 thrombolytic drugs (tenecteplase and urokinase) showed a nonsignificant difference between groups (OR = 1.13, 95% CI = 0.83–1.53, *p* = 0.43; Supplementary Fig S6C). In the TICI 2c‐3 subgroup pooling data from 5 anterior circulation RCTs, a stratified meta‐analysis by thrombolytic drug revealed varying efficacy. Alteplase demonstrated a significant benefit (OR = 2.95, 95% CI = 1.60–5.41, *p* < 0.001), whereas tenecteplase (OR = 1.11, 95% CI = 0.75–1.65, *p* = 0.59) and urokinase (OR = 1.22, 95% CI = 0.87–1.72, *p* = 0.25) a nonsignificant difference between groups. The overall meta‐analysis across all thrombolytic drugs suggested a nonsignificant difference between groups (OR = 1.45, 95% CI = 0.96–2.20, *p* = 0.08), with significant heterogeneity among subgroups (*p* = 0.02; Supplementary Fig S6D).

### Subgroup Analysis of IVT before EVT

Two trials (CHOICE and PEARL), which included 122 IVT‐treated patients, were pooled for excellent functional outcome (mRS = 0–1). The overall pooled OR was 1.33 (95% CI = 0.75–2.36), which was not statistically significant (*p* = 0.33; Supplementary Fig S7).

## Functional Independence (mRS = 0–2) at 90 Days

**FIGURE 3:**
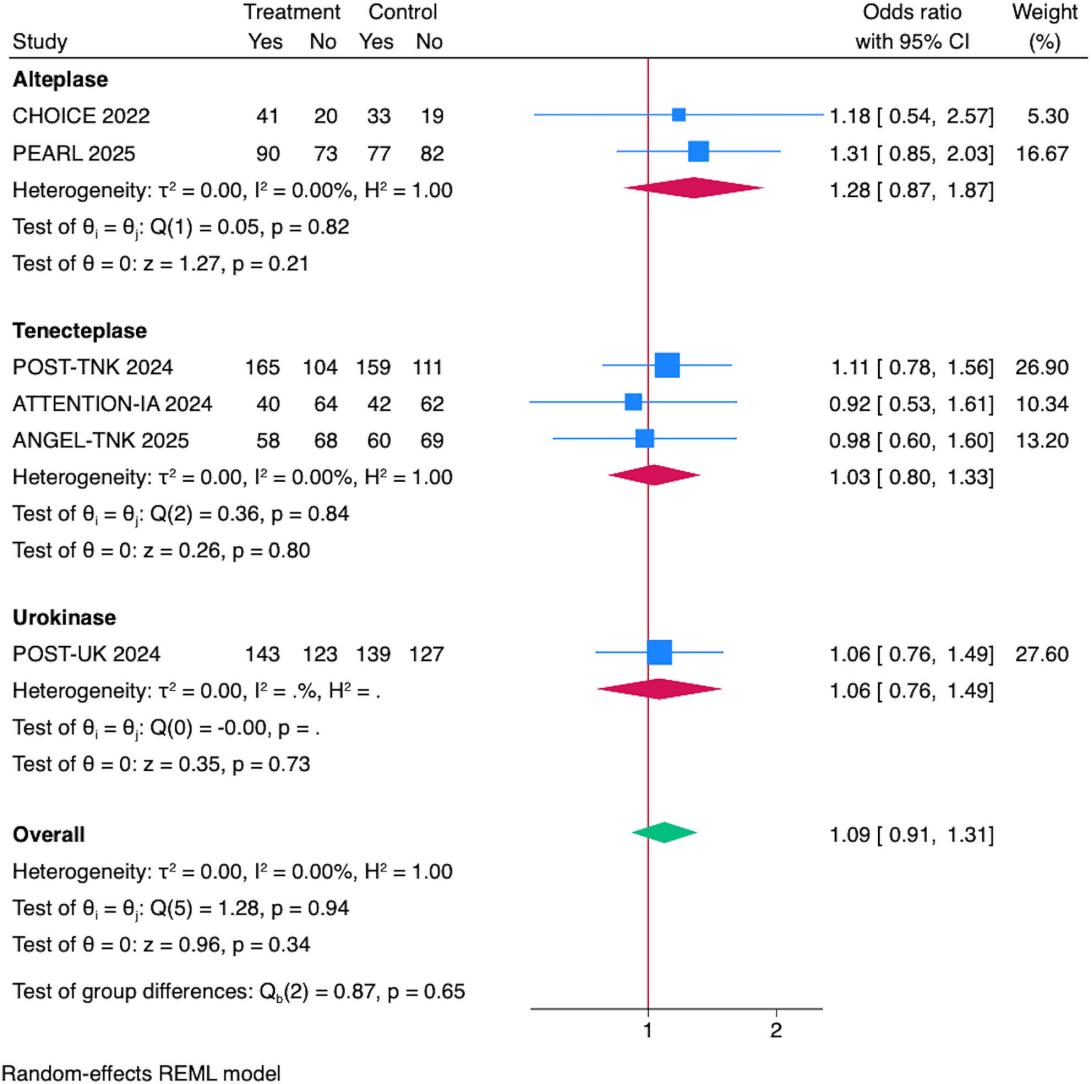
Association between IAT and functional independence (mRS = 0–2) at 90 days. Pooled analysis showed no significant improvement in 90‐day mRS = 0–2 with IAT (OR = 1.09, 95% CI = 0.91–1.31, *p* = 0.30). Subgroup analysis by drug showed no benefit with alteplase (OR = 1.28, 95% CI = 0.87–1.87, *p* = 0.21), tenecteplase (OR = 1.03, 95% CI = 0.80–1.33, *p* = 0.77), or urokinase (OR = 1.06, 95% CI = 0.76–1.49, *p* = 0.73). CI = confidence interval; IAT = intra‐arterial thrombolysis; mRS = modified Rankin Scale; OR = odds ratio. [Color figure can be viewed at www.annalsofneurology.org]

In terms of functional independence (mRS = 0–2) at 90 days, IAT was not associated with increased odds of 90‐day mRS = 0–2 (OR = 1.09, 95% CI = 0.91–1.31, *p* = 0.30; Fig 3). Sensitivity analyses, including leave‐one‐out evaluations, confirmed the robustness of the results, with no single trial disproportionately influencing the pooled effect size or heterogeneity (see Supplementary Fig S2B). Sensitivity analyses excluding RCTs presented at ISC 2025 and including data from the DATE trial (phase IIa) confirmed the robustness of the results (see Supplementary Figs S3B and S4B).

### Subgroup Analyses by IAT Drug Type and Stroke Location

A subgroup analyses based on the type of IAT drug (alteplase: OR = 1.28, 95% CI = 0.87–1.87, *p* = 0.21; tenecteplase: OR = 1.03, 95% CI = 0.80–1.33, *p* = 0.77; urokinase: OR = 1.06, 95% CI = 0.76–1.49, *p* = 0.73; see Fig 3), and stroke location (anterior circulation: OR = 1.11, 95% CI = 0.92–1.34, *p* = 0.31, posterior circulation: OR = 0.92, 95% CI = 0.53–1.61, *p* = 0.77) did not show a significant benefit for functional independence (mRS = 0–2) at 90 days. Heterogeneity was low across all subgroups (*I* ^2^ = 0%; Supplementary Fig S8).

## Symptomatic Intracranial Hemorrhage

**FIGURE 4:**
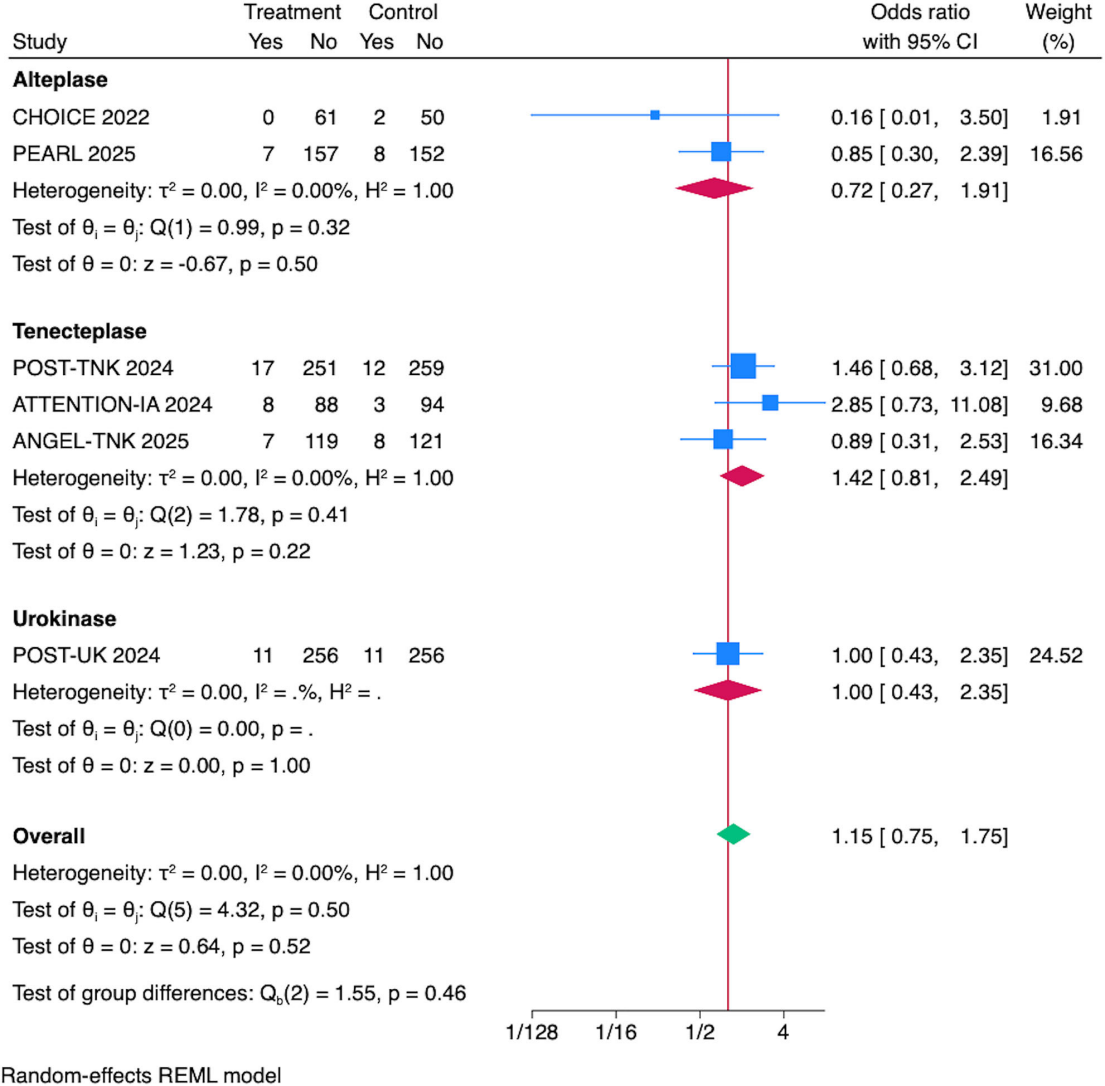
Association between IAT and sICH. The pooled OR for sICH across all studies was 1.15 (95% CI = 0.75–1.76, *p* = 0.51). Stratified by IAT drug, no significant association was found for alteplase (OR = 0.72, 95% CI = 0.27–1.92, *p* = 0.51), tenecteplase (OR = 1.42, 95% CI = 0.81–2.41, *p* = 0.22), or urokinase (OR = 1.00, 95% CI = 0.43–2.35, *p* > 0.999). No heterogeneity was observed (*I* ^2^ = 0%). CI = confidence interval; IAT = intra‐arterial thrombolysis; OR = odds ratio; sICH = symptomatic intracranial hemorrhage. [Color figure can be viewed at www.annalsofneurology.org]

The pooled OR for sICH across all studies was 1.15 (95% CI = 0.75–1.76, *p* = 0.51), showing that IAT was not associated with increased odds of sICH (Fig 4). Sensitivity analyses, including leave‐one‐out evaluations, confirmed the robustness of the results, with no single trial disproportionately influencing the pooled effect size or heterogeneity (see Supplementary Fig S2C). Sensitivity analyses excluding RCTs presented at ISC 2025 confirmed the robustness of the results (see Supplementary Fig S3C).

### Subgroup Analyses by IAT Drug Type and Stroke Location

**FIGURE 5:**
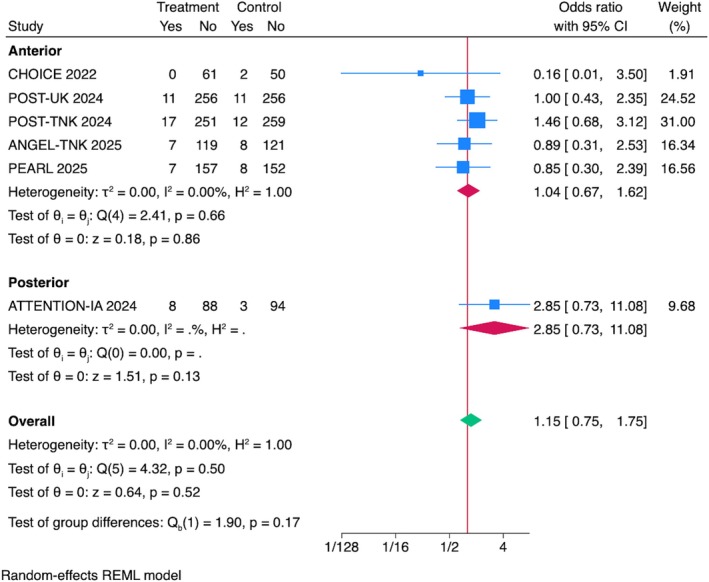
Association between IAT and sICH. The pooled OR for sICH across all studies was 1.15 (95% CI = 0.75–1.76, *p* = 0.51). Stratified by stroke location, no significant association was seen for anterior circulation (OR = 1.04, 95% CI = 0.67–1.63, *p* = 0.85) or posterior circulation strokes (OR = 2.85, 95% CI = 0.73–11.08, *p* = 0.13), with no heterogeneity (*I* ^2^ = 0%). CI = confidence interval; IAT = intra‐arterial thrombolysis; OR = odds ratio; sICH = symptomatic intracranial hemorrhage. [Color figure can be viewed at www.annalsofneurology.org]

When stratified by IAT drug, the odds of sICH were as follows: alteplase OR = 0.72, 95% CI = 0.27–1.92, *p* = 0.51; tenecteplase OR = 1.42, 95% CI = 0.81–2.41, *p* = 0.22, and urokinase OR = 1.00, 95% CI = 0.43–2.35, *p* > 0.999. No heterogeneity was observed within any subgroup (*I* ^2^ = 0.00%; see Fig 4). There was no significant association of IAT with sICH in subgroup analysis stratified by stroke location. The pooled OR for anterior circulation strokes was 1.04, 95% CI = 0.67–1.63, *p* = 0.85, whereas for posterior circulation strokes it was 2.85, 95% CI = 0.73–11.08, *p* = 0.13, but with no observed heterogeneity (*I* ^2^ = 0.00%; Fig 5).

## Mortality at 90 Days

**FIGURE 6:**
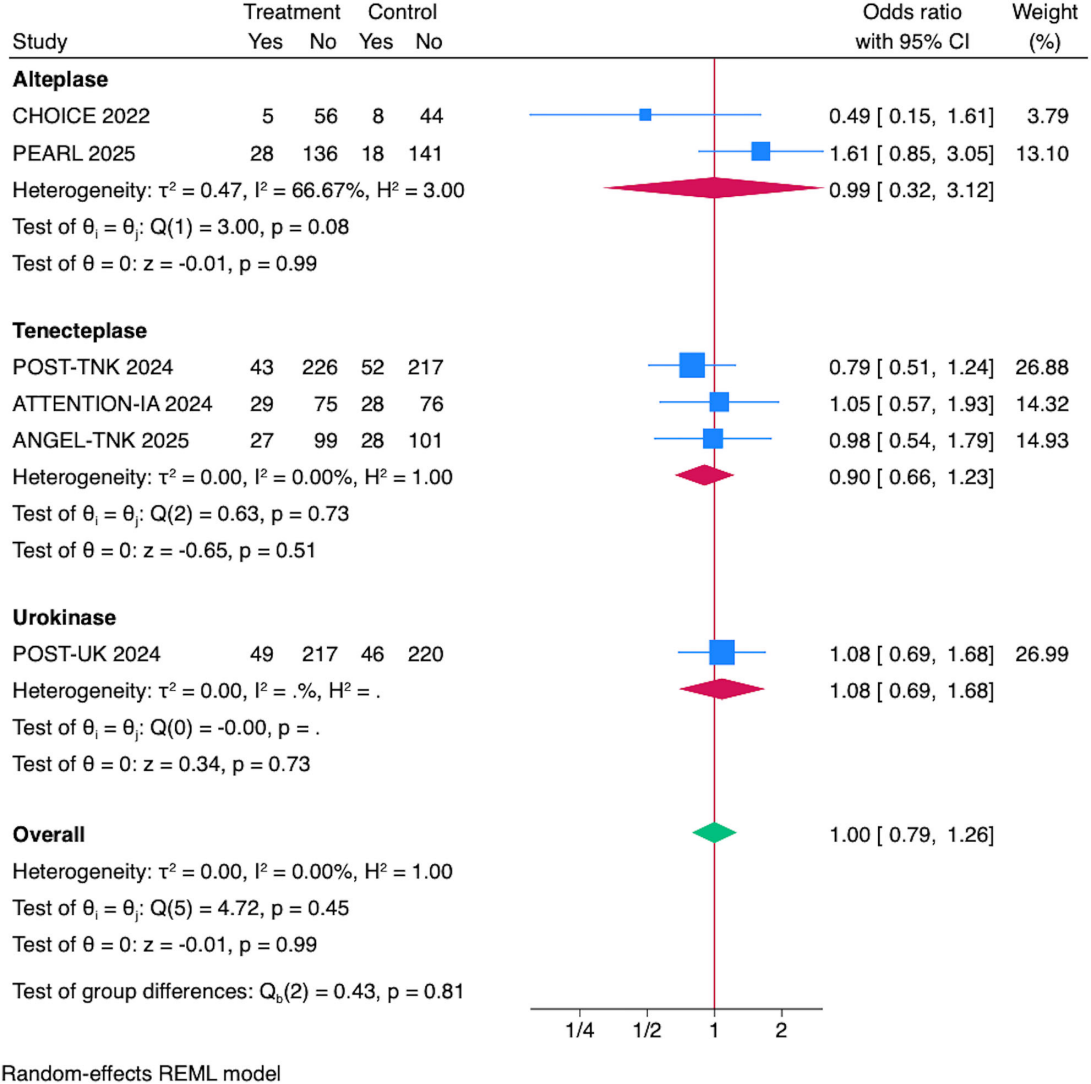
Association between IAT and mortality. Pooled analysis of 6 RCTs showed no significant difference in mortality between the IAT and the control groups (OR = 1.00, 95% CI = 0.79–1.26, *p* = 0.99). Subgroup analysis by IAT drug showed no mortality reduction with alteplase (OR = 0.99, 95% CI = 0.32–3.12, *p* = 0.99), tenecteplase (OR = 0.90, 95% CI = 0.66–1.23, *p* = 0.53), or urokinase (OR = 1.08, 95% CI = 0.69–1.68, *p* = 0.74). CI = confidence interval; IAT = intra‐arterial thrombolysis; OR = odds ratio; RCTs = randomized controlled trials. [Color figure can be viewed at www.annalsofneurology.org]

The overall pooled estimate showed no significant difference in mortality between the IAT and control group (OR = 1.00, 95% CI = 0.79–1.26, *p* = 0.99; Fig 6). Sensitivity analyses, including leave‐one‐out evaluations, confirmed the robustness of the results, with no single trial disproportionately influencing the pooled effect size or heterogeneity (see Supplementary Fig S2D). Sensitivity analyses excluding RCTs presented at ISC 2025 and including data from the DATE trial (phase IIa) confirmed the robustness of the results (see Supplementary Figs S3D and S4C).

### Subgroup Analyses by IAT Drug Type and Stroke Location

**FIGURE 7:**
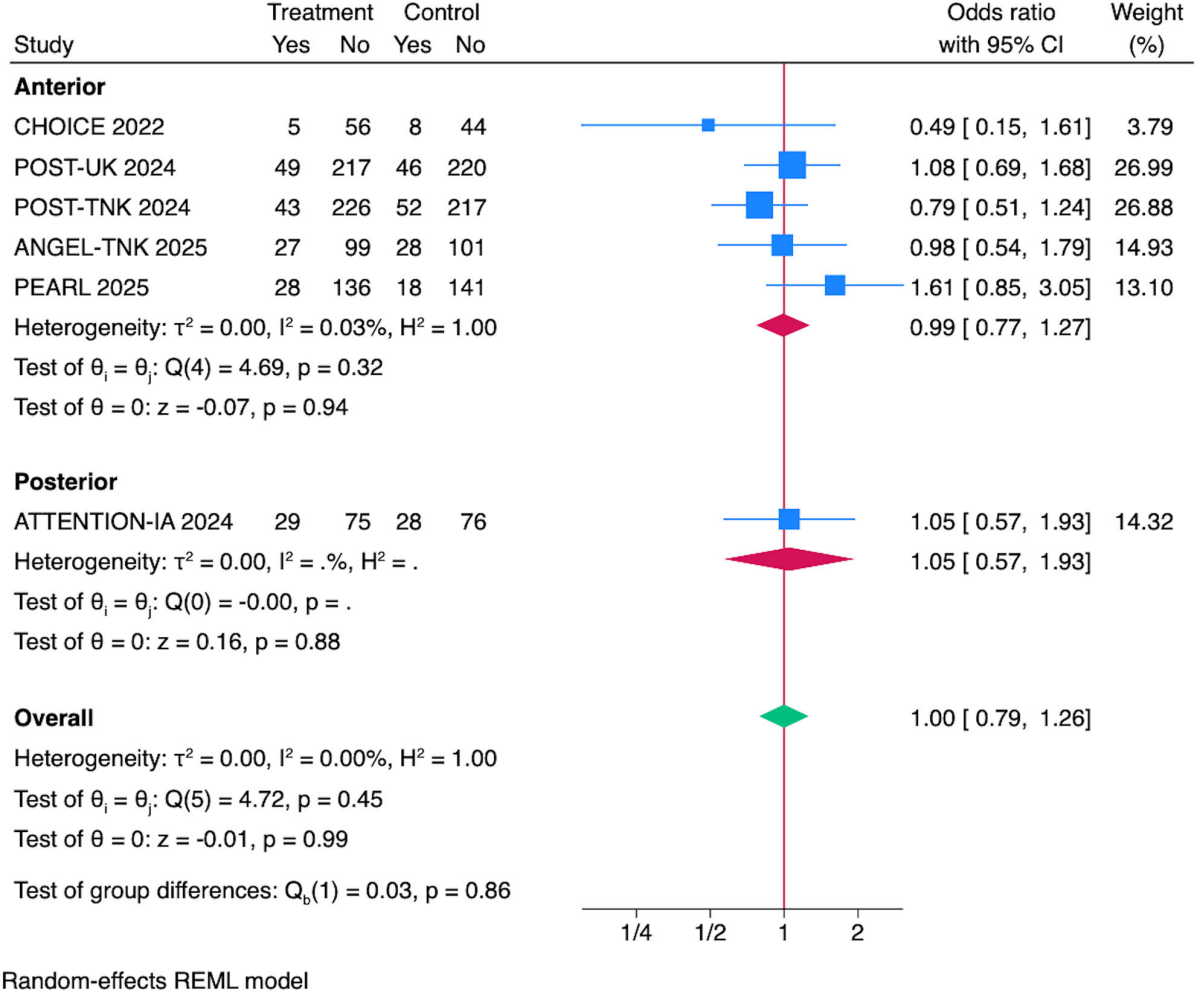
Association between IAT and mortality. Pooled analysis of 6 RCTs showed no significant difference in mortality between the IAT and the control groups (OR = 1.00, 95% CI = 0.79–1.26, *p* = 0.99). Subgroup analyses revealed no difference for anterior circulation strokes (OR = 0.99, 95% CI = 0.77–1.27, *p* = 0.94) or posterior circulation strokes (OR = 1.05, 95% CI = 0.57–1.93, *p* = 0.87), with no heterogeneity across subgroups (*I* ^2^ = 0%). CI = confidence interval; IAT = intra‐arterial thrombolysis; OR = odds ratio; RCTs = randomized controlled trials. [Color figure can be viewed at www.annalsofneurology.org]

Subgroup analysis by IAT drug demonstrated no significant mortality reduction with alteplase (OR = 0.99, 95% CI = 0.32–3.12, *p* = 0.99), tenecteplase (OR = 0.90, 95% CI = 0.66–1.23, *p* = 0.53), or urokinase (OR = 1.08, 95% CI = 0.69–1.68, *p* = 0.74; see Fig 6). Similarly, subgroup analysis by stroke location revealed no mortality difference in anterior circulation stroke (OR = 0.99, 95% CI = 0.77–1.27, *p* = 0.94) or posterior circulation stroke (OR = 1.05, 95% CI = 0.57–1.93, *p* = 0.87). There was no heterogeneity across subgroups (*I* ^2^ = 0.00%; Fig 7).

## Discussion

Our findings demonstrate that IAT after successful EVT significantly and safely improved the likelihood of achieving excellent functional outcomes (mRS = 0–1) at 90 days, particularly for patients with anterior circulation stroke. However, this benefit was equivocal in patients who suffered a posterior circulation stroke, given that the analysis only included one RCT and observed numerically higher rates of mRS = 0–1 favoring IAT after successful EVT (34.6% vs 26.0%). In addition to pooled trials, several ongoing trials are actively investigating the role of adjunctive IAT following successful recanalization with EVT. In the posterior circulation, the INSIST‐IT (NCT05657457) and ARTERIAL TNK BAO (NCT05580822) trials are evaluating the use of tenecteplase, whereas the IAT‐TOP trial (NCT05897554) is assessing alteplase. Meanwhile, in the anterior circulation, the CHOICE‐II trial (NCT05797792) is studying alteplase, and the EXTEND‐AGNES TNK trial (NCT05892510) is exploring tenecteplase. These studies aim to further clarify the potential benefits of IAT in improving outcomes after EVT and will provide more evidence.

The results of our study can be understood in the context of the IAT impact on the macro‐ and microcirculation. On one hand, for microcirculation, the observed improvement in outcomes for anterior circulation strokes can be attributed to the complementary effect of IAT in enhancing microvascular reperfusion “no reflow phenomenon.” The no‐reflow phenomenon, characterized by the lack of microvascular reperfusion despite successful macrovascular reperfusion, is common in LVOS. In a meta‐analysis of 13 studies, the no‐reflow phenomenon was observed in approximately one‐third of the patient with stroke who achieved successful macrovascular reperfusion (29%, 95% CI = 21%–37%). The no‐reflow phenomenon was also linked to poorer functional outcomes.^9^ However, its definition varied across studies, and it remains unclear whether no‐reflow is a result of persistent vessel occlusions or a separate pathological process.^9^ EVT is effective at removing the primary occlusion, but persistent microthrombi or incomplete tissue reperfusion can still limit recovery despite successful recanalization.^9^, ^10^ IAT may target these microthrombi, improving distal perfusion and tissue recovery. On the other hand, the overall positive results observed in TICI 2b‐3 trials, compared to those in TICI 2c‐3, may suggest a beneficial impact on macrocirculation by improving reperfusion grading. In contrast, the potential impact on microcirculation is better understood within the context of TICI 2c‐3 or TICI 3, where directional effects toward benefit were observed in our meta‐analysis, but the sample size was insufficient for statistical significance. Notably, excluding the ANGEL‐TNK trial, the pooled analysis demonstrated a significant benefit. The ANGEL‐TNK trial presented a treatment effect estimate that diverged from the overall trend observed in other included studies in terms of the proportion of patients with TICI 2c/3 reperfusion at randomization and achieving mRS = 0–1 (30.8% in the IAT group vs 38.0% in the EVT‐alone group). This discrepancy introduced potential bias and had the potential to distort the pooled effect estimate. To fully comprehend the microcirculatory effects, trials focusing exclusively on TICI 3 outcomes are needed. Additionally, it would be valuable to capitalize on macrocirculatory effects by prospectively assessing the role of IAT alone as a bailout strategy in cases of unsuccessful reperfusion (TICI = 0–2a) after the first pass with EVT, comparing it to continuing with additional passes. In this context, the TECNO trial (NCT05499832) is investigating whether IAT with tenecteplase can enhance early and late reperfusion in patients with incomplete reperfusion with EVT. Notably, the Adjunctive Intraarterial Tenecteplase Following Mechanical Thrombectomy (ALLY) Pilot Trial explored the feasibility and safety of using intra‐arterial tenecteplase in patients with incomplete recanalization following EVT. This prospective, single‐center, non‐randomized study demonstrated that intra‐arterial tenecteplase (up to 4.5 mg) was feasible and did not increase the incidence of ICH or neurological deterioration compared to EVT alone.^30^

Furthermore, exploring the role of IAT alone in distal or medium vessel occlusions (MeVOs) could be a promising area of research, especially given the recent negative RCTs examining EVT in this patient group.^31^, ^32^ For MeVOs, ongoing trials are exploring the role of adjunctive rescue treatments following successful proximal recanalization in LVOS. The 2BE3 trial (NCT06034847) aims to assess whether rescue therapy for persistent distal occlusions can improve outcomes. Patients will receive either mechanical intervention using small stent retrievers or aspiration catheters or pharmacological treatment with IAT. Meanwhile, the RESCUE TNK trial (NCT05657470) is evaluating the use of intra‐arterial tenecteplase in patients with primary MeVO, detected on the first DSA examination, or secondary MeVO following EVT for LVO. Notably, the ALLY Pilot Trial explored the feasibility and safety of using intra‐arterial tenecteplase in patients with incomplete recanalization following EVT. This prospective, single‐center, non‐randomized study demonstrated that intra‐arterial tenecteplase (up to 4.5 mg) was feasible and did not increase the incidence of ICH or neurological deterioration compared to EVT alone.^30^

Previous meta‐analyses, primarily based on observational studies and often using IAT as a rescue strategy for failed EVT rather than as an adjunct, have shown inconsistent results regarding IAT efficacy.^13^, ^16^ These studies frequently lacked key distinctions, such as whether successful reperfusion was achieved before IAT or if IAT was used as a rescue or adjunct therapy. Rescue IAT aims to achieve sufficient reperfusion when initial treatments fail, whereas adjunct IAT enhances microcirculation after partial or complete reperfusion. This ambiguity complicates reliable analyses, leading to potential biases and misinterpretations. Unlike other trials that included patients, eTICI 2b50‐3, the POST‐UK^17^, and POST‐TNK^18^ trials focused solely on eTICI 2c‐3; to better understand the role of IAT on micro‐ rather than macro‐perfusion. However, all trials used IAT as an adjunct after successful recanalization. Across the RCTs included in this meta‐analysis (CHOICE, POST‐TNK, POST‐UK, ANGEL‐TNK, ATTENTION‐IA, PEARL, and DATE), IAT was administered following successful endovascular thrombectomy, defined as eTICI 2b–3 or 2c–3 reperfusion. Although the protocols varied slightly, several core elements were consistent and may have influenced treatment effects. CHOICE and PEARL used intra‐arterial alteplase (0.225 mg/kg), whereas POST‐TNK, ATTENTION‐IA, ANGEL‐TNK, and DATE used tenecteplase at doses ranging from 0.03125 to 0.125 mg/kg; POST‐UK utilized intra‐arterial urokinase (see the Table 1). IAT was consistently initiated immediately after angiographic confirmation of reperfusion. Delivery should generally target distal emboli or areas of impaired microcirculatory flow, with attention to residual thrombus burden and proximal perforators. Angiographic assessment can help guide catheter positioning and ensure drug delivery to the intended vascular territory. These methodological variations, although subtle, highlight the importance of procedural details in interpreting efficacy across studies.

It is noteworthy that sICH and mortality outcomes were robust across various subgroup analyses. The odds of sICH did not significantly differ by thrombolytic drug or stroke location, confirming a favorable safety profile for IAT with no observed heterogeneity. Similarly, there was no significant difference in 90‐day mortality between IAT and control groups, regardless of drug or stroke location. Sensitivity analyses further affirmed the stability of these findings. These results support the safety of IAT while demonstrating its efficacy in improving stroke outcomes. Further studies are needed to explore the optimal thrombolytic drug, dosing regimen, and time window for IAT, which may vary depending on the type of stroke and the patient's clinical condition. The phase Ib/IIa DATE trial results on dose escalation of adjunctive tenecteplase following successful EVT suggest that doses of 0.03125 or 0.0625 mg/kg were safe.^29^ Interestingly, we observed numerically higher odds of sICH in posterior circulation strokes compared with anterior circulation strokes (OR = 1.04, 95% CI = 0.67–1.63 vs 2.85, 95% CI = 0.73–11.08). Although no significant heterogeneity was detected in our analysis, the sample size for posterior circulation strokes was small, and the findings were derived from a single trial. Prior studies have suggested differences in the hemorrhagic risks of endovascular intervention across anterior and posterior circulations.^33^, ^34^

Two trials (CHOICE and PEARL) involving IVT‐treated patients were combined, revealing no significant difference were observed in excellent functional outcome (mRS = 0–1) between both groups. This finding contrasts with the positive effects seen in the overall meta‐analysis. The discrepancy may be due to the limited statistical power of this subgroup analysis, which included only 2 trials, or the increased risk of bleeding associated with IVT before EVT, potentially reducing the advantages of IAT. Additionally, IVT may enhance reperfusion grading and improve outcomes, which could limit the added benefit of IAT in this context. The IRIS meta‐analysis demonstrated that for final reperfusion, EVT alone resulted in lower eTICI scores compared to IVT combined with EVT (eTICI score 2b–3: 921/1,093, 84.3% vs 973/1,101, 88.4%; adjusted OR [aOR] = 0.62, 95% CI = 0.45–0.86, *p* = 0.0038; eTICI score 2c–3: 587/1,093, 53.7% vs 638/1,101, 57.9%; aOR = 0.83, 95% CI = 0.67–1.03, *p* = 0.094).^35^ Furthermore, early recanalization, defined as the absence of treatable occlusion or reperfusion eTICI 2b–3, was lower in EVT alone compared to IVT plus EVT (19/1,108, 1.7% vs 45/1,125, 4.0%; aOR = 0.41, 95% CI = 0.18–0.92, *p* = 0.031).^35^ Further research is needed to clarify the interaction between IVT before EVT and adjunctive IAT, particularly regarding their impact on reperfusion and functional outcomes, in larger and adequately powered studies.

Although our meta‐analysis demonstrated a statistically significant benefit of adjunctive IAT in achieving excellent functional outcomes (mRS = 0–1), the effect was less pronounced and did not reach statistical significance for the broader category of good outcomes (mRS = 0–2). Therefore, our results should be cautiously interpreted. This may be attributed to plausible mechanistic factors such as enhanced microvascular reperfusion and reduction of distal emboli, which could selectively improve the quality of recovery, shifting patients from mRS = 2 to 1 or 0 without substantially impacting the overall rate of good outcomes. Notably, prior randomized trials of IVT have similarly demonstrated a benefit in terms of excellent outcomes (mRS = 0–1) but failed to reach statistical significance for functional independence (mRS = 0–2).^36^, ^37^

To address potential concerns regarding the inclusion of unpublished data, we ensured that all such datasets were validated by the lead investigators of the respective trials (ANGEL‐ASPECT, PEARL, and DATE), who participated in this pooled analysis. Only raw event counts for each outcome were included, which are not subject to change during peer review. These data were cross‐checked against both conference presentations (ISC 2025) and final manuscript submissions. Sensitivity analyses excluding conference‐only data yielded consistent results, confirming the robustness of our findings. Furthermore, we conducted a leave‐one‐out analysis to ensure that no single study disproportionately influenced the overall results. This methodological rigor strengthens confidence in the reliability and generalizability of our conclusions, despite the inclusion of yet‐to‐be‐published trials. Despite the promising results of this meta‐analysis, several limitations should be acknowledged. Methodological heterogeneity across studies, including variations in IAT drug and patient selection, may have influenced the findings. Although the random‐effects model accounted for this heterogeneity, these variations still pose limitations. Additionally, whereas sensitivity analyses were performed to assess the robustness of the results, the small number of studies in some subgroups, particularly based on stroke location, may have limited the ability to detect significant differences. Future research should focus on identifying specific patient subgroups most likely to benefit from IAT, with advanced imaging techniques such as perfusion imaging playing a crucial role in patient selection. Larger, more uniform RCTs are needed to confirm the optimal use of IAT in conjunction with EVT, particularly for TICI 3.

In conclusion, our systematic review and meta‐analysis suggest that IAT can significantly improve functional outcomes after successful EVT for patients with anterior circulation LVOS, without increasing the risk of sICH or mortality. However, the risks and benefits in posterior circulation strokes remain unclear, and further research is needed to determine the optimal patient population for this adjunctive therapy. Despite the positive findings, the variability in study design and the mixed results from prior studies highlight the need for additional high‐quality trials to establish the role of IAT in modern stroke care. As we continue to refine our treatment strategies, patient selection and individualized approaches will be critical to maximizing the benefits of IAT in the context of EVT.

## Author Contributions

M.F.D., M.H.M., and R.G.N. contributed to the conception and design of the study; M.F.D., R.G.N., A.J., and M.H.M. contributed to the acquisition and analysis of data; M.F.D., M.H.M., A.J., Q.Y., W.Z., Y.C., Y.T., Y.L., X.H., L.L., B.Y., Z.M., W.H., C.T., X.L., L.J., X.B., W.C., D.C.H., T.N., and R.G.N. contributed to drafting the text and preparing the figures.

## Potential Conflicts of Interest

The authors report no conflicts of interest relevant to this paper.

## Supporting information


**Data S1.** Supporting Information.

## Data Availability

All data included in this paper are available in the paper itself and the supplementary materials.
