# Supplementary material for: Adjunctive Thrombolytics After Successful Endovascular Reperfusion: A Systematic Review and Meta‐Analysis of Randomized Controlled Trials
Source: Ann Neurol. 2025 Aug 13;98(6):1299–314. doi: 10.1002/ana.70021 (PMC12682943; doi:10.1002/ana.70021)
Supplement: Supplementary file 1 — Data S1. Supporting Information. [file ANA-98-1299-s001.docx]

**SUPPLEMENT**

**Figure S1. Flow diagram of the search process**


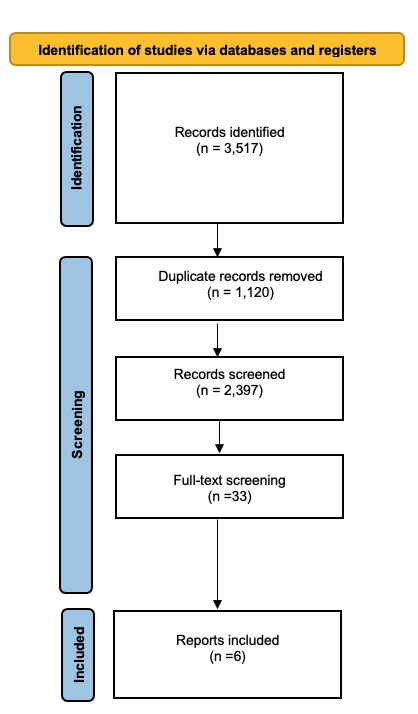


**Figure S2 shows the results of a leave-one-out analysis for various outcomes. Panel A presents the analysis for mRS 0-1 (excellent functional outcome), while Panel B displays mRS 0-2 (favorable functional outcome). Panel C shows symptomatic intracranial hemorrhage (sICH) mortality outcomes, Panel D focuses on mortality at 90 days**

**Figure S3. Pooled Subgroup Analysis of IVT Before Endovascular Thrombectomy**

**Figure S3. Sensitivity analysis excluding RCTs presented during ISC 2025.**

1. mRS 0-1 at 90 days

1. mRS 0-2 at 90 days

1. SICH

1. Death at 90 days

**Figure S4. All RCTs, including DATE (phase 2a).**

1. mRS 0-1:

1. mRS 0-2:

1. Death at 90 days:

**Table S1. Results of quality assessment using the Cochrane collaboration’s tool for randomized controlled trials.**

| Study | Randomization process | Deviation from the intended intervention | Missing outcomes | Measurement of the outcome | Selection of the reported results | Overall |
| --- | --- | --- | --- | --- | --- | --- |
| CHOICE 2022 | Low | Low | Low | Low | Low | Low |
| POST-UK 2024 | Low | Low | Low | Low | Low | Low |
| POST-TNK 2024 | Low | Low | Low | Low | Low | Low |
| ATTENTION-IA 2024 | Low | Low | Low | Low | Low | Low |
| ANGEL-TNK 2025 | Low | Low | Low | Low | Low | Low |
| PEARL 2025 | Low | Low | Low | Low | Low | Low |

# **Table S2. Quality of Evidence Summary**

| Certainty Assessment | mRS 0–1 at 90 Days | mRS 0–2 at 90 Days | Symptomatic Intracranial Hemorrhage | Mortality at 90 Days |
| --- | --- | --- | --- | --- |
| Number of Studies | 6 | 6 | 6 | 6 |
| Study Design | Randomized Trial | Randomized Trial | Randomized Trial | Randomized Trial |
| Risk of Bias | Not Serious | Not Serious | Not Serious | Not Serious |
| Inconsistency | Not Serious | Not Serious | Not Serious | Not Serious |
| Indirectness | Not Serious | Not Serious | Not Serious | Not Serious |
| Imprecision | Not Serious | Not Serious | Not Serious | Not Serious |
| Other Considerations | None | None | None | None |
| Effect Estimate 95% CI) | 1.23 (1.11–1.36) | 1.04 (0.96–1.13) | 1.23 (0.81–1.85) | 0.98 (0.82–1.18) |
| Certainty of Evidence | ⨁⨁⨁⨁ High | ⨁⨁⨁⨁ High | ⨁⨁⨁⨁ High | ⨁⨁⨁⨁ High |
